# Ceftriaxone Calcium Crystals Induce Acute Kidney Injury by NLRP3-Mediated Inflammation and Oxidative Stress Injury

**DOI:** 10.1155/2020/6428498

**Published:** 2020-07-09

**Authors:** Zhang Yifan, Ning Benxiang, Xu Zheng, Xu Luwei, Zhou Liuhua, Ge Yuzheng, Jia Ruipeng

**Affiliations:** ^1^Department of Urology, Nanjing First Hospital, Nanjing Medical University, 68 Changle Road, Nanjing 210006, China; ^2^Nanjing Drum Tower Hospital, The Affiliated Hospital of Nanjing University Medical School, No. 321, Zhongshan Road, Nanjing 210000, China

## Abstract

**Objective:**

To investigate the role of inflammatory reactions and oxidative stress injury in the mechanisms of ceftriaxone calcium crystal-induced acute kidney injury (AKI) both in vivo and in vitro.

**Methods:**

Male Sprague Dawley rats were randomly divided into five groups of ten each according to different concentrations of ceftriaxone and calcium. Based on the levels of serum creatinine (Scr) and blood urea nitrogen (BUN), the AKI group was chosen for the subsequent experiments. Kidney histological examination and immunohistochemistry were performed. The expression of NLRP3 and IL-1*β* protein and the concentrations of oxidative stress markers such as ROS, MDA, and H_2_O_2_ in kidney tissues were estimated. In parallel, HK-2 human renal proximal tubule cells were exposed to ceftriaxone calcium crystals. The mRNA expression levels of NLRP3 and IL-1*β* and the concentrations of oxidative stress markers were evaluated. Finally, cell viability and rat survival were also assessed.

**Results:**

The results showed that significantly increased Scr and BUN levels, consistent with morphological changes and kidney stones, were found in the rats that received the highest concentration of ceftriaxone (1000 mg/kg) combined with calcium (800 mg/kg). The activation of the NLRP3 inflammasome axis and the marked elevation of MDA, H_2_O_2_, and ROS levels were observed both in vivo and in vitro. High expression of Nrf2, HO-1, and NQO1 was also documented. In addition, cell apoptosis and rat mortality were promoted by ceftriaxone calcium crystals.

**Conclusions:**

Notably, we found that ceftriaxone-induced urolithiasis was associated with a high risk of AKI and NLRP3-mediated inflammasome and oxidative stress injury were of major importance in the pathogenesis.

## 1. Introduction

Ceftriaxone, a potent, semisynthetic, third-generation cephalosporin has a wide spectrum of powerful antimicrobial activities. The intravenous administration of ceftriaxone has been widely used for the treatment of microbial infections, particularly organ infection and sepsis [1]. Ceftriaxone is highly soluble as a sodium salt. However, it can bind with calcium ions, producing a poorly soluble ceftriaxone–calcium salt that forms precipitates in the urinary tract, also known as urolithiasis [2]. Although its incidence is relatively rare, ceftriaxone-induced urolithiasis could lead to severe complications, such as acute kidney injury (AKI) [3]. Based on our previous systematic review, the proportion of ceftriaxone calculi-induced AKI was 72.7%, which was much higher than the proportion of AKI induced by other types of stones, including 9.8% for melamine stones and only a rare occurrence for calcium oxalate stones [4]. The traditional concept of crystal-induced kidney injury focuses on urinary tract obstruction. Undoubtedly, bilateral obstructive urolithiasis can cause acute kidney injury, but tubular crystal plugs and casts rarely obstructed enough nephrons at the same time to explain AKI [5]. Our previous study also found that in addition to urinary obstruction, which is well-known, crystalline nephropathy could contribute to ceftriaxone calculi-induced AKI [4].

The evolving evidence that toxic and postischemic AKIs are largely driven by the associated inflammatory response raised the question of whether inflammation was also the driving factor in crystal-induced AKI [6]. Recently, it has been shown that CaOx crystals activated the NLRP3 inflammasome, resulting in progressive renal failure [7]. Other studies illustrated that cystine crystals, similar to CaOx, were endogenous inflammasome-activating stimuli [8]. These discoveries on the molecular mechanisms of crystal-induced inflammation now enforce a new view on crystal-related kidney injury. The most thoroughly described inflammasome is the nucleotide-binding domain and leucine-rich repeat protein-3 (NLRP3) [9–11]. NLRP3 assembles a multiprotein complex termed inflammasome, which comprises a caspase recruitment domain (Asc), and induces caspase-1 activation and the maturation of proinflammatory cytokines such as IL-1*β* and IL-18 [9]. The NLRP3 inflammasome is the best studied among all inflammasomes [10]; however, in ceftriaxone calcium crystal-induced AKI, the role of NLRP3 has yet not been researched.

Numerous studies have indicated that oxidative stress can play a significant role in the development of kidney stones [12, 13]. In spite of urolithiasis, acute kidney injury was also associated with ROS production and impaired antioxidant activity [14]. In murine-accelerated, severe lupus nephritis, the production of the inflammatory cytokine IL-18 could be reduced by enhancing antioxidant activation [15]. So based on analogy with other forms of crystal-induced kidney injury, we speculated that oxidative stress and NLRP3-mediated inflammation could both contribute to ceftriaxone calcium crystal-induced AKI. The purpose of this trial was to examine the role of NLRP3-mediated inflammation and oxidative stress injury in promoting the progressive renal failure observed in a model of ceftriaxone calcium crystal nephropathy.

## 2. Results

### 2.1. Rats Administered with High Concentration of Ceftriaxone and Calcium Developed AKI

**Figure 1:**
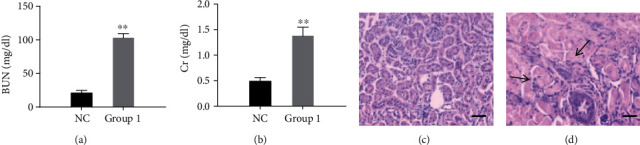
Rats administered with high concentration of ceftriaxone and calcium developed AKI. (a) Concentrations of blood urea nitrogen (mg/dL). (b) Concentrations of creatinine (mg/dL) (*n* = 10 for each group; the results are expressed as mean ± SD; ^∗∗^*P* < 0.01 compared with the control group). (c, d) Representative H&E-stained kidney sections ((c) NC group; (d) group 1, bars = 50 *μ*m). Injury scores were 1 and 4, respectively. The arrows indicated the representative morphological changes including severe interstitial edema, cellular infiltrate, tubular cell swelling, and necrosis.

We previously successfully created a rat model of ceftraxone-induced urolithiasis [4]. In the present trial, the rats were randomly divided into two groups. Compared with the rats in the NC group, the rats (group 1) that were administered with ceftriaxone (1000 mg/kg)+CaCl_2_ (800 mg/kg) revealed a severe increase in BUN (Figure 1(a)) and creatinine (Figure 1(b)), consistent with renal failure (*P* < 0.01). H&E staining also showed histopathological damage to the kidney, including severe interstitial edema, cellular infiltrate, tubular cell swelling, and necrosis (Figures 1(c) and 1(d)).

### 2.2. High Concentration of Ceftriaxone and Calcium Induced Crystal Nephropathy and Inflammation

**Figure 2:**
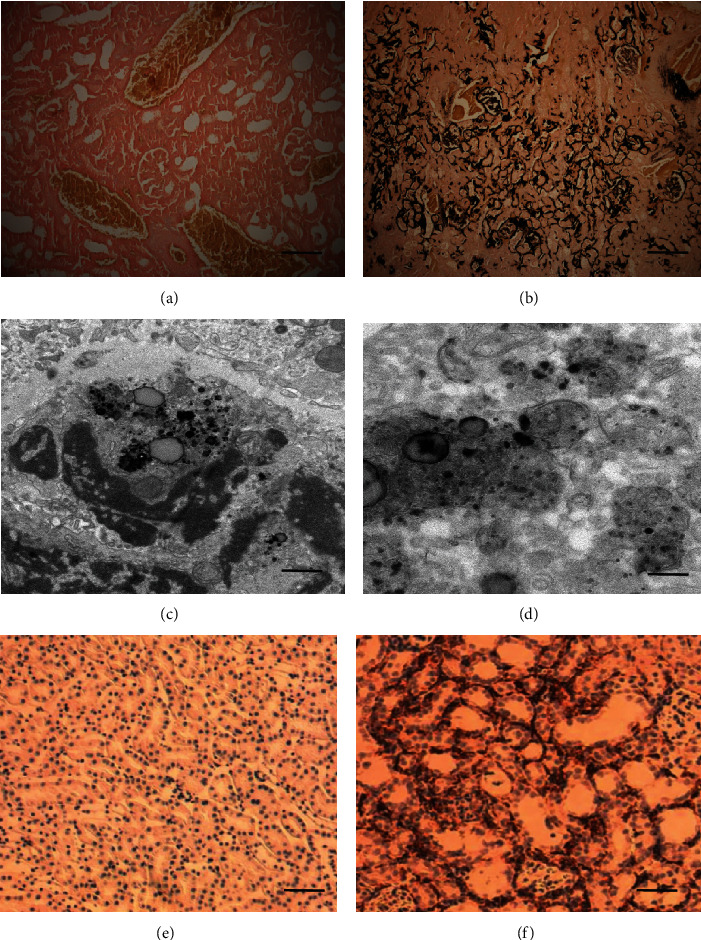
Rats administered with high concentration of ceftriaxone and calcium developed crystal nephropathy and inflammation. (a, b) Representative von Kossa staining of kidney sections ((a) NC group; (b) group 1, bars = 200 *μ*m). (c, d) TEM images of extracellular (c) and intracellular (d) crystal precipitates. (e, f) F4/80 staining for macrophages/monocytes of kidney sections ((e) NC group; (f) group 1, bars = 200 *μ*m).

As shown in Figure 2, histological examination using von Kossa staining (Figures 2(a) and 2(b)) demonstrated profound crystal deposition in the kidneys of rats administered with ceftriaxone and calcium. The expansion of the kidney tubules was also observed. Transmission electron microscopy (TEM) showed that there were both intracellular and extracellular crystal precipitates (Figures 2(c) and 2(d)). To further define the inflammatory infiltrate, we stained for the presence of macrophages/monocytes with immunohistochemistry. As indicated in Figures 2(e) and 2(f), macrophage/monocyte staining was strongly positive in the group treated with ceftriaxone and calcium compared with the control group.

### 2.3. Ceftriaxone Calcium Crystals Upregulated the NLRP3 Inflammasome Axis

**Figure 3:**
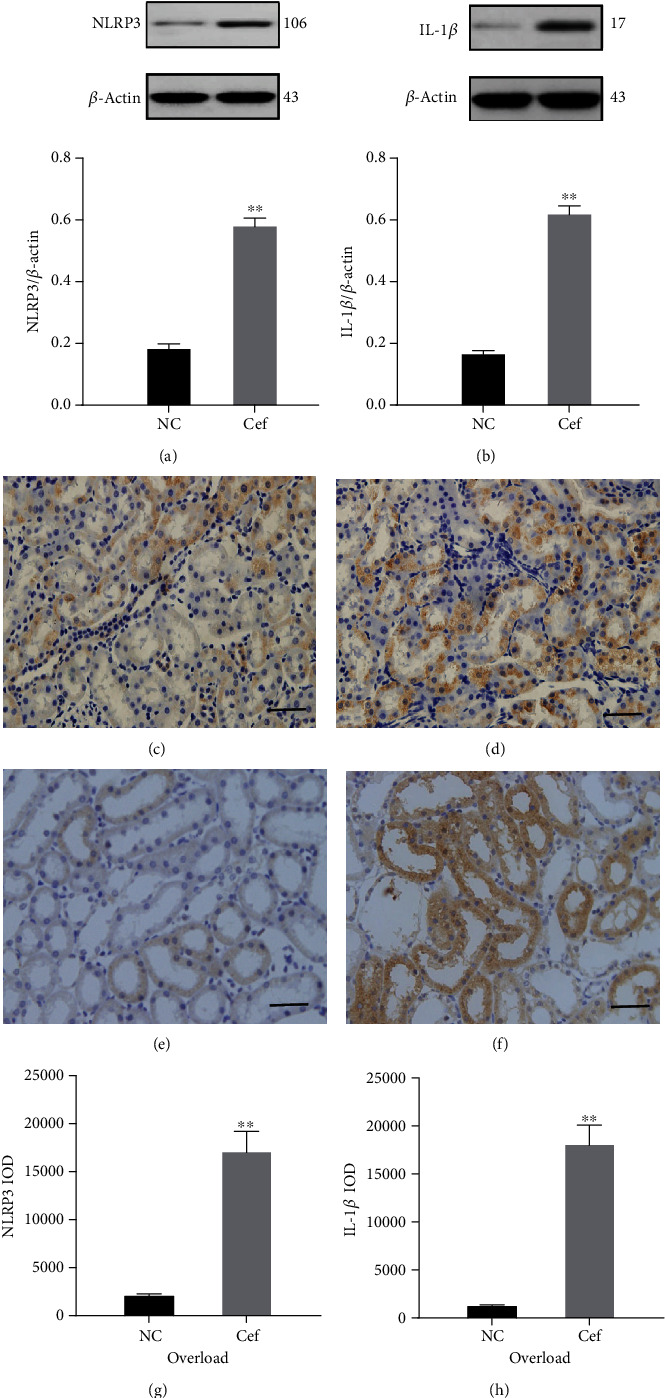
Ceftriaxone calcium crystals upregulated the NLRP3 inflammasome axis. (a, b) Representative western blots and quantitative analysis for NLRP3 (a) and IL-1*β* (b) expression in the kidney tissues. *β*-Actin was used as the internal control. (c, d) Immunohistochemistry staining of NLRP3 in kidney tissue ((c) NC group, (d) group 1, bars = 100 *μ*m). (e, f) Immunohistochemistry staining of IL-1*β* in kidney tissue ((e) NC group, (f) group 1, bars = 100 *μ*m). (g, h) Semiquantitative analysis for NLRP3 (g) and IL-1*β* (h) using Image-Pro plus 6.0 software (the results are expressed as mean ± SD; ^∗∗^*P* < 0.01 compared with the control group).

We measured the expression of NLRP3 and IL-1*β* in the kidney lysates, and the western blot results demonstrated that they were noticeably elevated in the AKI group (Figures 3(a) and 3(b)). In addition, immunohistochemical staining also showed that NLRP3 and IL-1*β* were upregulated in the tubules of rats in the AKI group compared with those in the control group (Figures 3(c)–3(h)).

### 2.4. Ceftriaxone Calcium Crystals Increased Oxidative Stress

**Figure 4:**
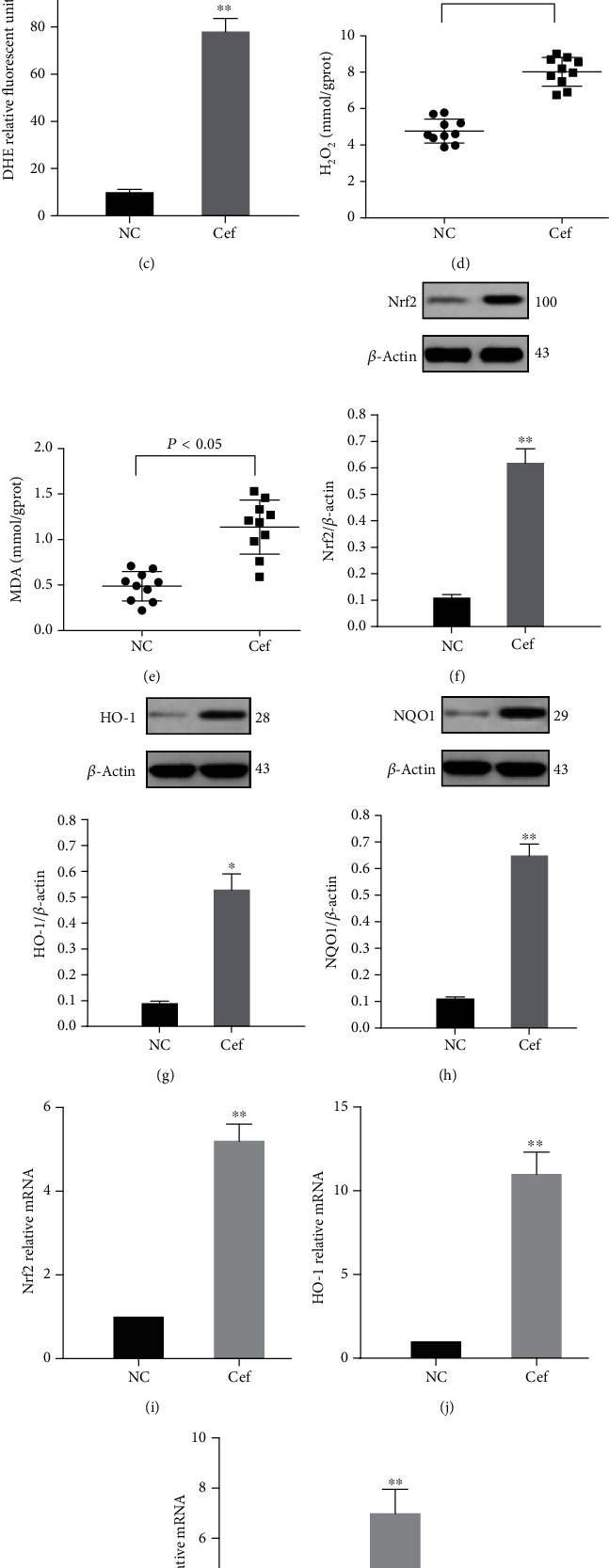
Ceftriaxone calcium crystals increased oxidative stress. (a, b) DHE fluorescence in the kidney tissue ((a) NC group, (b) group 1, bars = 100 *μ*m). (c) Graph representing mean DHE fluorescence. (d) Concentrations of H_2_O_2_ in the kidney tissue. (e) Concentrations of MDA in the kidney tissue. (f–h) Representative western blots and quantitative analysis for Nrf2 (f), HO-1 (g), and NQO1 (h) expression in the kidney tissues. *β*-Actin was used as the internal control. (i–k,) mRNA levels of Nrf2 (i), HO-1 (j), and NQO1 (k) in vivo (the results are expressed as mean ± SD; ^∗^*P* < 0.05 compared with the control group, ^∗∗^*P* < 0.01 compared with the control group).

ROS production was increased in the renal proximal tubules of rats, as assessed by ROS-sensitive vital dye DHE (Figures 4(a)–4(c)). Furthermore, the H_2_O_2_ and MDA concentrations of rats were measured (Figures 4(d) and 4(e)). The results showed that the H_2_O_2_ and MDA concentrations in the kidney tissues were significantly increased in the AKI group compared with those in the control group. Next, we investigated whether ceftriaxone calcium crystals affected the Nrf2 pathway in the kidneys of rats with AKI. As shown in Figures 4(f)–4(h), the results of western blot analysis revealed that the expression of Nrf2 including its downstream enzymes such as HO-1 and NQO1 were enhanced. Consistent with the protein analyses, Nrf2, HO-1, and NQO1 mRNA levels were also found to be markedly elevated (Figures 4(i)–4(k)).

### 2.5. Ceftriaxone Calcium Crystals Promoted NLRP3 Inflammasome Activation and Oxidative Stress Injury In Vitro

**Figure 5:**
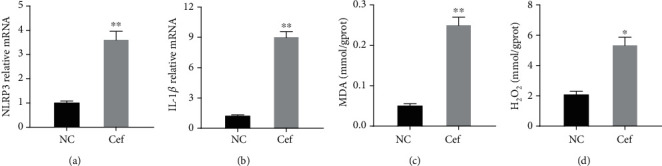
Ceftriaxone calcium crystals promoted NLRP3 inflammasome activation and oxidative stress injury in vitro. (a, b) mRNA levels of NLRP3 (a) and IL-1*β* (b) in vitro. (c) Concentrations of MDA in vitro. (d) Concentrations of H_2_O2 in vitro (the results are expressed as mean ± SD; ^∗^*P* < 0.05 compared with the control group, ^∗∗^*P* < 0.01 compared with the control group).

By applying quantitative RT-PCR, we examined the mRNA expression levels of NLRP3 and IL-1*β* in HK-2 cells treated with ceftraxone calcium crystals. Obviously, NLRP3 and IL-1*β* mRNA levels were remarkably induced in the treatment group compared with the control group (Figures 5(a) and 5(b)). Moreover, the H_2_O_2_ and MDA concentrations of HK-2 cells were also examined. The treatment of HK-2 cells with ceftriaxone calcium crystals significantly increased H_2_O_2_ and MDA concentrations (Figures 5(c) and 5(d)).

### 2.6. Ceftriaxone Calcium Crystals Induced Cell Apoptosis and Rat Mortality

**Figure 6:**
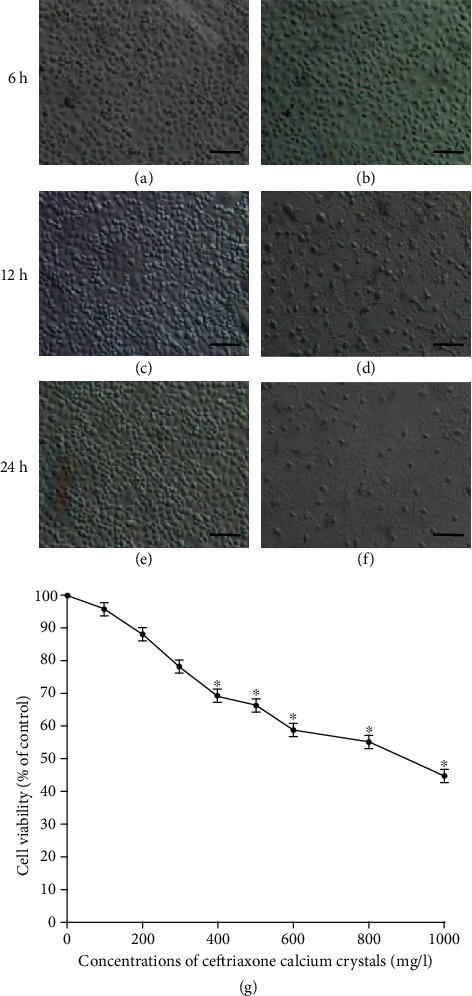
Ceftriaxone calcium crystals induced cell apoptosis. (a, c, e) SEM images of the control group for 6, 12, and 24 h. (b, d, f) HK-2 cells were treated with ceftriaxone calcium crystals (500 *μ*g/mL) for 6, 12, and 24 hours, and SEM images were documented (bars = 1 *μ*m). (g) Dose-dependent effects of ceftriaxone calcium crystals on HK-2 cell viability (the results are expressed as mean ± SD; ^∗^*P* < 0.05 compared with the control group).

**Figure 7:**
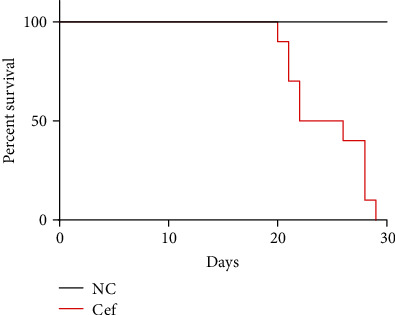
Ceftriaxone calcium crystals induced rat mortality. Rats administered with high concentration of ceftriaxone and calcium demonstrated 100% mortality at 30 days. In contrast, the control group resulted in 100% survival at 30 days. Data are expressed as Kaplan-Meier survival curve.

As shown in Figure 6, scanning electron microscopy (SEM) images of the control group showed normal healthy morphology with minimal apoptosis (Figures 6(a), 6(c), and 6(e)). In contrast, ceftriaxone calcium crystal treatment for 24 h induced massive apoptosis with cellular and nuclear condensation and fragmentation (Figure 6(f)). The dose-dependent effects of ceftriaxone calcium crystals (0–1000 mg/L) on HK-2 cell viability (Figure 6(g)) showed that a ceftriaxone crystal concentration ≥ 500 mg/L was capable of significantly reducing cell viability (*P* < 0.05). To examine the effect of ceftriaxone calcium calculi-induced AKI on rat mortality, we continued to observe the NC group and AKI group for 30 days. The 30-day mortality of the AKI group was 100% (Figure 7). Death likely occurred following progressive renal insufficiency, but the contribution of inflammation in other organ systems cannot be excluded. In contrast, the NC group showed 100% survival at 30 days.

### 2.7. Effects of MCC950 on Cell Viability and Cytoprotection

**Figure 8:**
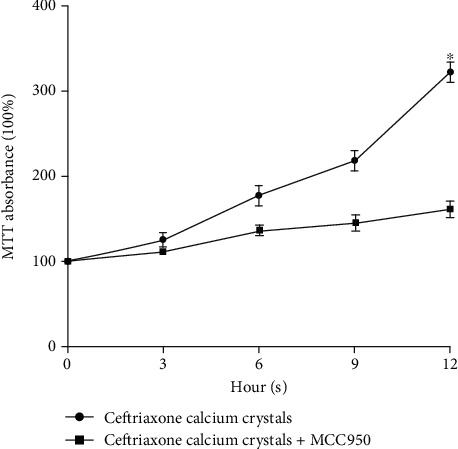
Effects of MCC950 on ceftriaxone calcium crystal-induced cytotoxicity. HK-2 cells were treated with ceftriaxone calcium crystals alone and in combination with MCC950 (10 *μ*M) for 3, 6, 9, and 12 hours, and MTT absorbance was examined (the results are expressed as mean ± SD; ^∗^*P* < 0.05 compared with the control group).

MCC950, the inhibitor of NLRP3, was added to HK-2 cell cultures containing ceftriaxone calcium crystals for 12 hours, after which they were subjected to MTT assays. The results (Figure 8) showed that the ceftriaxone calcium crystal-treated cells had significantly increased MTT absorbance levels, which were prevented by the addition of MCC950.

## 3. Discussion

Recently, AKI was found secondary to ceftriaxone-induced urolithiasis [16, 17]. Our previous study also demonstrated impaired renal function in 72.7% of the patients with ceftriaxone calculi [4]. Renal stone disease is widely considered to be a mechanical problem causing intrarenal or extrarenal urinary outflow obstruction, but crystal nephropathy is also associated with significant intrarenal inflammation and oxidative stress that can cause acute kidney injury [5]. In the present study, our findings suggested that the inflammasome and oxidative stress injury were of major importance in the loss of kidney function that occurred in ceftriaxone calcium crystal-induced kidney disease, an important example of crystal nephropathy.

The inflammasome is thought to have the unique ability to integrate a myriad of signals from pathogen- and damage-associated molecular patterns into a proinflammatory response [18]. As the most extensively investigated of the inflammasomes identified, the activation of the NLRP3 inflammasome can be achieved by a wide range of structurally dissimilar agonists, including pathogens, pore-forming toxins, environmental irritants, and endogenous damage-associated molecular patterns [19–21]. Our study found that NLRP3 activation was involved in the pathogenesis of ceftriaxone calcium crystal-induced AKI and cell apoptosis. And the inhibitor of NLRP3 could effectively protect renal cells from ceftriaxone attack, suggesting that ceftriaxone crystal-mediated cytotoxicity was likely associated with NLRP3 inflammasome. In fact, NLRP3 activation has been demonstrated to contribute to multiple diseases by increasing IL-1*β* and IL-18 secretion and amplifying the inflammatory response, including acute lung injury and acute liver failure [22, 23]. Evolving data suggest that the inflammasome IL-1/18 axis contributes to acute and chronic inflammation and tissue remodeling in the kidney [5]. Our study defined the relative importance of the inflammasome in specific ceftriaxone calcium crystal-induced AKI and suggested further study to identify the therapeutic opportunities afforded by targeting the inflammasome response.

Oxidative stress has been implicated in the pathogenesis of AKI [24]. In the present study, markers of oxidative stress such as ROS, MDA, and H_2_O_2_ were significantly increased in the animal model of ceftriaxone calculi-induced AKI, and similar changes were also observed in HK-2 cells. As reported, Nrf2 is a pivotal coordinator of the pathways that protects cells against oxidative and inflammatory damage, with HO-1 and NQO1 being two of the major downstream anti-inflammatory and antioxidative enzymes [25]. Nrf2 translocation from the cytoplasm to the nucleus was essential for its regulation of antioxidant/detoxification enzyme expression [26]. We showed here that ceftriaxone calcium crystal stimulation increased Nrf2 levels in the nuclei, as well as HO-1 and NQO1 protein and mRNA levels, which was indicative of a stress response. Our results were also consistent with those of Zhang et al., who demonstrated that Nrf2 played a protective role in acute lung injury in vitro and in vivo [27].

In addition to the indirect mechanisms involving inflammation and oxidative stress injury that we have discussed, ceftriaxone calcium crystals could also harm tubular cells by directly inducing tubular cell death. Crystals are known to be cytotoxic when they come in direct contact with the tubular epithelium [28]. For example, Thongboonkerd et al. have demonstrated that CaOx monohydrate crystals (COM) tightly adhere to MDCK cells with subsequent detrimental effects on the cells [29]. The internalization of COM crystals by MDCK cells resulted in the alteration of several proteins involved in energy production, which contributed to mitochondrial dysfunction and a subsequent increase in ROS production [30]. We have shown that upon exposure, ceftriaxone calcium crystals could directly induce massive apoptosis, consistent with the cellular and nuclear condensation and fragmentation of HK-2 cells. H&E staining also showed severe interstitial edema, cellular infiltrate, tubular cell swelling, and necrosis. Taken together, these results indicated that the pathological changes promoted the elevation of Cr and BUN levels and the subsequent progressive renal insufficiency, which eventually induced rat mortality.

There is limited information about ceftriaxone-induced urolithiasis and its association with AKI. This severe complication is often overlooked or misinterpreted, although its incidence is extremely high. To our knowledge, this is the first study to investigate its pathogenesis, and the present study revealed several new findings. First, we developed an animal model of ceftriaxone calcium crystal nephropathy with development of renal failure and death. Second, we demonstrated that inflammation and oxidative stress played a critical role in the mechanism of ceftriaxone calcium crystal-induced AKI. The necroptosis of renal tubules and cell apoptosis were consistent with the increased expression of IL-1*β* and concentrations of ROS, MDA, and H_2_O_2_, suggesting that inflammation and oxidative stress could promote renal injury. Finally, we highlighted a novel role of NLRP3 and Nrf2 in these events. Based on our study, ceftriaxone calcium crystals significantly elevated the expression of the NLPR3-IL-1*β* axis and the transcription of Nrf2, including the downstream enzymes, such as HO-1 and NQO1. Collectively, these results suggested that ceftriaxone calcium crystals could induce acute kidney injury by NLRP3-mediated inflammation and oxidative stress injury. Further research is necessary to determine the therapeutic value of blocking of the inflammasome pathway consistent with antioxidants.

The current study still has several limitations. First, the present trial lacked positive and negative controls to further verify the role of NLRP3 and Nrf2 in the pathogenesis of ceftriaxone calcium crystal-induced AKI. For example, a series of measures such as NLRP3 inhibitor, si-NLRP3 cells, NLRP3 knockout rats, and si-Nrf2 cells could be used to make the results more convincing. Second, as reported, ROS production could also lead to the activation of the NLRP3 inflammasome [20]. Thus, the effect of oxidative stress on increasing the expression of the NLRP3 axis cannot be ruled out, and whether ceftriaxone calcium crystals could have a direct effect on NLRP3 inflammasome activation is not clear. Further studies should be performed to determine the in-depth mechanisms.

## 4. Conclusion

In summary, we have developed an animal model of ceftriaxone calcium crystal nephropathy with development of renal failure and death. Using this model, we demonstrated that NLRP3-mediated inflammasome and oxidative stress injury are of major importance in the pathogenesis of ceftriaxone calcium crystal-induced AKI. This work emphasized the potential value of anti-inflammatory and antioxidant therapies in ceftriaxone calcium crystal nephropathy.

## 5. Methods

### 5.1. Animal Experiment

The animal experiment procedure consisted of two steps.

*Step1*. A total of 20 male SD rats, initially weighing 100.0 ± 5.1 g, were housed under standard conditions (the animal experimental center of Nanjing Medical University, SCXK 2008-0004). All of the rats were given free access to deionized distilled water throughout the experiment. This experiment was permitted by the Veterinary Directorate of Nanjing Medical University, in accordance with the Chinese legislation and the Council Directive of the Asian Community.

The rats were randomly divided into two groups (ten rats each) as follows: (1) NC group, no treatment, and (2) group 1, ceftriaxone (1000 mg/kg)+CaCl_2_ (800 mg/kg). Ceftriaxone was administered by intramuscular injections, while CaCl_2_ was administered by gavage. All of the reagents were administered for 7 days.

On day 7, all the rats were anesthetized with ketamine (100 mg/kg), and blood samples (3 mL each) were collected by cardiac puncture to assay the Cr (creatinine) and BUN (blood urea nitrogen) concentrations. All of the collected samples were kept at -20°C until measurements were taken. Then, the rats were sacrificed, and their kidneys were removed for histological examination (the procedure was described in Section 5.2).

*Step2*. The AKI group was rebuilt for the next experiments. The amount of living rats was documented, and the 30-day survival rates were calculated.

### 5.2. Renal Histology

The kidney tissues were fixed with paraformaldehyde (4%) and, after 48 hours, paraffin-embedded and sectioned into 4 *μ*m thick, and then, some were processed for hematoxylin and eosin (H&E) staining and evaluated using a light microscope (Olympus BX40, Tokyo, Japan). Others were stained with von Kossa staining and were scanned using polarization microscopy with a Nikon TE2000U inverted microscope and MetaMorph software (Scion Inc., USA). Quantification of ceftriaxone calcium crystal deposition was performed by scanning whole kidneys under polarized light and setting a pixel intensity threshold that identifies crystals separate from background tissue. Ultrastructural analysis was performed with a transmission electron microscope EM 410 (Philips; Eindhoven, Netherlands). An avidin-biotin immunoperoxidase method was used with a monoclonal antibody directed against macrophages/monocytes (F4/80; clone BM8, Abcam, no. 16911). The injury scores were graded according to the degree of damage based on the percentage of involvement of the kidney. The damage quantification from ten areas corresponding to the renal tissue was graded using the following parameters: tubular cell necrosis, apoptosis, cytoplasmic vacuole formation, hemorrhage, and tubular dilatation based on a five-score system (1, histopathological changes < 10%; 2, =10-25%; 3, =25-50%; 4, =50-75%; and 5, =75-100%) [31]. The pathological scores were given by two independent blinded pathologists.

### 5.3. Cell Experiments

The human kidney-2 cells (HK-2) were purchased from HonorGene company (Changsha, China). They were cultured in Eagle's minimum essential medium supplemented with 10% fetal bovine serum (Huihe, Guangzhou), penicillin (100 U/mL, Rongbo, Shanghai), and streptomycin (100 *μ*g/mL, Kairui, Beijing). For experiments, cells were seeded in six-well plates or T-75 flasks at the initial cell density of 2 × 10^5^ cells/mL and divided into a control group and ceftraxone calcium crystal-treated group. The MDA (malondialdehyde) and H_2_O_2_ concentrations were measured, and the mRNA expression levels of NLRP3 and IL-1*β* were examined (the methods were described in Sections 5.5 and 5.8). The specimens were sputter-coated with platinum and examined by JEOL JSM-6500 TFE-SEM at an accelerating voltage of 10 keV.

### 5.4. Western Blot Analysis

After cell lysates were obtained from control and agent-treated cells, approximately 40 *μ*g proteins were subjected to 10% SDS–page and transferred to nitrocellulose membrane. Membranes were blocked at room temperature for 1 h in blocking buffer containing 5% nonfat dry milk to prevent nonspecific binding and then incubated with primary antibodies overnight at 4°C. The primary antibodies used in the present study was anti-Nrf2 (Biorbyt, Cambridge, 1 : 200), anti-NLRP3 (MyBioSource, LLC, San Diego, California, 1 : 1000, anti-IL-1*β* (ab-9722, Abcam, 1 : 1000), anti-HO-1 (Bioss Antibodies, Beijing, 1 : 1,000), and Anti-NQO1 (Bioss Antibodies, 1 : 1,000). The membranes were washed in TBST (50 mmol/L Tris-HCl, pH 7.6; 150 mmol/L NaCl; 0.1% Tween 20) for 30 min and incubated with an appropriate secondary antibody (Sigma, 1 : 2000 dilution). Bound antibody was visualized with a commercial enhanced chemiluminescence kit (Amersham Pharmacia Biotech) and Kodak film. The level of each protein was expressed relative to the amount of actin.

### 5.5. Measurement of the MDA and H_2_O_2_ Concentrations

The kidney tissues of rats were collected as described above. MDA and H_2_O_2_ concentrations of kidney tissues and HK-2 cells were measured with colorimetric detection kits (Biorbyt, Wuhan, China).

### 5.6. Measurement of Reactive Oxygen Species (ROS) Level

Fresh kidney tissues were mounted using Tissue-Tek O.C.T. Unfixed frozen samples were cut into 5 *μ*m thick sections and placed on glass slides. DHE (dihydroethidium, 10 *μ*mol/L) was applied to each tissue section, and then, the slides were incubated in a light-protected humidified chamber at 37°C for 30 min. The resulting color reaction was immediately measured with a fluorescence microscopy (Leica, Germany) and analyzed by image densitometry using ImageJ software (NIH).

### 5.7. Immunohistochemistry

4 *μ*m thick kidney sections embedded in paraffin were prepared for immunohistochemistry assays. Sections were rehydrated, and antigens retrieved using heated citrate. Primary antibodies including NLPR3 (Adipogen, USA) and IL-1*β* (Miltenyi, China) were applied in blocking solution overnight at 4°C. After being incubated with anti-rabbit IgG (1 : 100; Beyotime) for 30 min at 37°C and washed and developed with DAB (Bioss, China), the stained sections were examined using a light microscope (Nikon Eclipse TE2000-U, NIKON, Japan) at 400x magnification. The semiquantitative immunohistochemical analysis was scored using Image-Pro plus 6.0 software in ten randomly selected cortical sections in each tissue section.

### 5.8. qPCR (Quantitative Polymerase Chain Reaction)

Total renal RNA was isolated from the kidney of individual mice using TRI Reagent RNA Isolation Reagent (Sigma-Aldrich, St. Louis, MO, USA) according to the manufacturer's instructions. Real-time qPCR was performed using an ABI PRISM 7300 Real-Time PCR System (Applied Biosystems, USA). The primer sequences for quantitative qPCR were as follows: NLRP3, 5′-CCACAGTGTAACTTGCAGAAGC-3′ (forward) and 5′-GGTGTGTGAAGTTCTGGTTGG-3′ (reverse); Nrf2, 5′-TCTCCTCGCTGGAAAAAGAA-3′ (forward) and 5′-AATGTGCTGGCTGTGCTTTA-3′ (reverse); HO-1, 5′-ATCGTGCTCGCATGAAC-3′ (forward) and 5′-CAGCTCCTCAAACAGCTCAA-3′ (reverse); NQO1, 5′-CAGCGGCTCCATGTACT-3′ (forward) and 5′-GACCTGGAAGCCACAGAAG-3′ (reverse); IL-1*β*, 5′-CCTCGTGCTGTCGGACCCATA-3′ (forward) and 5′-CAGGCTTGTGCTCTGCTTGTGA-3′ (reverse); and GAPDH, 5′-GGCATGGACTGTGGTCATGA-3′ (forward) and 5′-TTCACCACCATGGAGAAGGC-3′ (reverse). The gene expression from each sample was analyzed in duplicate and normalized against the internal control gene GAPDH.

### 5.9. Cell Viability Test

After treatment with various concentrations of ceftriaxone calcium crystals (0-1000 mg/L), the cell viability was then determined at the specified times using the MTT-based toxicology assay, following the manufacturer's protocol (Sigma, Shanghai, China). Then, HK-2 cells were divided into 2 groups as follows: ceftriaxone calcium crystals (1000 mg/L) and ceftriaxone calcium crystals (1000 mg/L)+MCC950 (10 *μ*M, Jinhui Biotechnology, Shanghai). After 12 h treatment, cell viability was determined using the MTT-based toxicology assay, following the manufacturer's protocol (Sigma, Shanghai, China).

### 5.10. Statistical Analysis

All of the statistical analyses were conducted by SPSS (version 19.0). Discrete variables were represented as frequencies, and the differences were evaluated by the Pearson *χ*^2^ test. Continuous variables were summarized as the mean ± standard deviation (SD), and the tests of significance were performed by *t*-tests for two groups or by an analysis of variance for more than two groups. A *P* value of less than 0.05 was considered significant.

## Data Availability

The data used to support the findings of this study are available from the corresponding author upon request.

## References

[1] Donnelly P. C., Sutich R. M., Easton R., Adejumo O. A., Lee T. A., Logan L. K. (2017). Ceftriaxone-associated biliary and cardiopulmonary adverse events in neonates: a systematic review of the literature. *Paediatric Drugs*.

[2] Cong X., Gu X., Sun X., Ning B., Shen L. (2014). Possible function of urinary pH and citrate on the ceftriaxone-induced nephrolithiasis. *Urology*.

[3] Tang X., Lieske J. C. (2014). Acute and chronic kidney injury in nephrolithiasis. *Current Opinion in Nephrology and Hypertension*.

[4] Zhang Y., Ning B., Zhu H. (2016). Characterizing ceftriaxone-induced urolithiasis and its associated acute kidney injury: an animal study and Chinese clinical systematic review. *International Urology and Nephrology*.

[5] Mulay S. R., Evan A., Anders H. J. (2014). Molecular mechanisms of crystal-related kidney inflammation and injury. Implications for cholesterol embolism, crystalline nephropathies and kidney stone disease. *Nephrology, Dialysis, Transplantation*.

[6] Kurts C., Panzer U., Anders H. J., Rees A. J. (2013). The immune system and kidney disease: basic concepts and clinical implications. *Nature Reviews. Immunology*.

[7] Knauf F., Asplin J. R., Granja I. (2013). NALP3-mediated inflammation is a principal cause of progressive renal failure in oxalate nephropathy. *Kidney International*.

[8] Prencipe G., Caiello I., Cherqui S. (2014). Inflammasome activation by cystine crystals: implications for the pathogenesis of cystinosis. *Journal of the American Society of Nephrology*.

[9] Liu Z., Wang X., Wang Y., Zhao M. (2017). NLRP3 inflammasome activation regulated by NF-*κ*B and DAPK contributed to paraquat-induced acute kidney injury. *Immunologic Research*.

[10] Darisipudi M. N., Knauf F. (2016). An update on the role of the inflammasomes in the pathogenesis of kidney diseases. *Pediatric Nephrology*.

[11] Leemans J. C., Kors L., Anders H. J., Florquin S. (2014). Pattern recognition receptors and the inflammasome in kidney disease. *Nature Reviews. Nephrology*.

[12] Ceban E., Banov P., Galescu A., Botnari V. (2016). Oxidative stress and antioxidant status in patients with complicated urolithiasis. *Journal of Medicine and Life*.

[13] Liang L., Li L., Tian J. (2014). Androgen receptor enhances kidney stone-CaOx crystal formation via modulation of oxalate biosynthesis & oxidative stress. *Molecular Endocrinology*.

[14] Shang Y., Siow Y. L., Isaak C. K., Karmin O. (2016). Downregulation of glutathione biosynthesis contributes to oxidative stress and liver dysfunction in acute kidney injury. *Oxidative Medicine and Cellular Longevity*.

[15] Tsai P. Y., Ka S. M., Chang J. M. (2012). Antroquinonol differentially modulates T cell activity and reduces interleukin-18 production, but enhances Nrf 2 activation, in murine accelerated severe lupus nephritis. *Arthritis and Rheumatism*.

[16] Li N., Zhou X., Yuan J., Chen G., Jiang H., Zhang W. (2014). Ceftriaxone and acute renal failure in children. *Pediatrics*.

[17] Shen X., Liu W., Fang X. (2014). Acute kidney injury caused by ceftriaxone-induced urolithiasis in children: a single-institutional experience in diagnosis, treatment and follow-up. *International Urology and Nephrology*.

[18] Wang G. L., Yuan H. M., Wang Z. F. (2018). Soluble uric acid activates NLRP3 inflammasome in myocardial cells through down-regulating UCP2. *Journal of Sichuan University Medical Science Edition*.

[19] Joshi S., Wang W., Peck A. B., Khan S. R. (2015). Activation of the NLRP3 inflammasome in association with calcium oxalate crystal induced reactive oxygen species in kidneys. *The Journal of Urology*.

[20] Tang T.-T., Lv L.-L., Pan M.-M. (2018). Hydroxychloroquine attenuates renal ischemia/reperfusion injury by inhibiting cathepsin mediated NLRP3 inflammasome activation. *Cell Death & Disease*.

[21] Braga T. T., Forni M. F., Correa-Costa M. (2017). Soluble uric acid activates the NLRP3 inflammasome. *Scientific Reports*.

[22] Zhao W. Y., Zhang L., Sui M. X., Zhu Y. H., Zeng L. (2016). Protective effects of sirtuin 3 in a murine model of sepsis-induced acute kidney injury. *Scientific Reports*.

[23] Shen J., Wang L., Jiang N. (2016). NLRP3 inflammasome mediates contrast media-induced acute kidney injury by regulating cell apoptosis. *Scientific Reports*.

[24] Feng X., Guan W., Zhao Y. (2019). Dexmedetomidine ameliorates lipopolysaccharide-induced acute kidney injury in rats by inhibiting inflammation and oxidative stress via the GSK-3*β*/Nrf 2 signaling pathway. *Journal of Cellular Physiology*.

[25] Mondal N. K., Saha H., Mukherjee B., Tyagi N., Ray M. R. (2018). Inflammation, oxidative stress, and higher expression levels of Nrf 2 and NQO1 proteins in the airways of women chronically exposed to biomass fuel smoke. *Molecular and Cellular Biochemistry*.

[26] Verma A. K., Yadav A., Singh S. V., Mishra P., Rath S. K. (2018). Isoniazid induces apoptosis: role of oxidative stress and inhibition of nuclear translocation of nuclear factor (erythroid-derived 2)-like 2 (Nrf 2). *Life Sciences*.

[27] Zhang H. X., Liu S. J., Tang X. L. (2016). H2S Attenuates LPS-induced acute lung injury by reducing oxidative/nitrative stress and inflammation. *Cellular Physiology and Biochemistry*.

[28] Peerapen P., Chaiyarit S., Thongboonkerd V. (2018). Protein network analysis and functional studies of calcium oxalate crystal-induced cytotoxicity in renal tubular epithelial cells. *Proteomics*.

[29] Thongboonkerd V., Semangoen T., Sinchaikul S., Chen S. T. (2008). Proteomic analysis of calcium oxalate monohydrate crystal-induced cytotoxicity in distal renal tubular cells. *Journal of Proteome Research*.

[30] Chaiyarit S., Thongboonkerd V. (2012). Changes in mitochondrial proteome of renal tubular cells induced by calcium oxalate monohydrate crystal adhesion and internalization are related to mitochondrial dysfunction. *Journal of Proteome Research*.

[31] Hu B., Tong F., Xu L. (2018). Role of calcium sensing receptor in streptozotocin-induced diabetic rats exposed to renal ischemia reperfusion injury. *Kidney & Blood Pressure Research*.

